# Posterior Circulation Stroke From Paradoxical Embolism in a Patient With Patent Foramen Ovale Dislodged During Masturbation

**DOI:** 10.1002/ccr3.73890

**Published:** 2026-11-06

**Authors:** Abdulmuti Saleh, Ashley Brown, Michael C. Larkins, Michael Tensmeyer, Florian Merkle

**Affiliations:** ^1^ Department of Emergency Medicine Boonshoft School of Medicine at Wright State University Fairborn Ohio USA; ^2^ Department of Clinical Neurosciences Boonshoft School of Medicine at Wright State University, Miami Valley Hospital Dayton Ohio USA

**Keywords:** patent foramen ovale, sexual behavior, stroke, Tenecteplase, young adult

## Abstract

Diagnosis of stroke in young and otherwise healthy individuals is rare, but early detection and treatment are crucial to patient outcomes. There may be unusual factors that lead to a presentation of stroke as seen in this case.

## 1 Introduction

Paradoxical embolism occurs when a thrombus passes from systemic venous circulation to the arterial circulation; one mechanism for this to happen is a patent foramen ovale (PFO). This allows a thrombus to bypass lung filtration and enter the arterial circulation through the atria [1]. Paradoxical emboli have been implicated in approximately 4% of ischemic strokes, rising to 55% among patients ≤ 60 years. Multiple theories exist regarding the etiology of the initial thrombus formation, including formation within the PFO versus accumulation of thrombus in the venous system as a pulmonary embolism or deep venous thrombus, which can fragment and pass through the PFO. Inciting factors for the development of a DVT and its subsequent embolization reported in the literature include trauma, thrombophilia, infection, and vigorous exercise, though notably not sexual activity [2, 3, 4]. As a potential exception, we present the case of a posterior circulation stroke in a 25‐year‐old patient during sexual activity and the acute management in the emergency department.

## 2 Case Presentation

A 25‐year‐old male presented to the Emergency Department (ED) via private vehicle, complaining of sudden‐onset numbness on the right side of his body, shortness of breath, and difficulty reading. He stated his symptoms came on while he was masturbating with his right hand and then persisted for 2 h, prompting his presentation to the ED. He was asymptomatic before symptom onset. He also reported an occipital headache that started 1 h after the numbness and vision changes. A few days prior, the patient traveled 2.5 h by car, but he reported no other recent travel. The patient reported a history of migraines without a formal diagnosis. He had multiple episodes of suspected transient ischemic attacks over the past 2 weeks in which he had “dark spots” in his vision, which occurred suddenly and resolved after a few minutes. These were followed by a posterior headache, which also self‐resolved after a few hours. The patient also gave a history of caffeine withdrawal headaches and typically drinks two to three cups per day. He had not had any caffeine on the day of presentation to the ED and initially attributed his occipital headache to caffeine withdrawal. He denied autoerotic asphyxiation and did not endorse any unusual or harmful behaviors during his masturbation. He also denied chest pain, fever, chills, night sweats, and taking any medications regularly. The patient's initial vital signs were unremarkable, with a blood pressure of 141/88. He was afebrile, maintaining adequate oxygen saturation on room air without tachypnea. He was generally well‐appearing on physical exam, with a benign abdominal and cardiovascular evaluation. Neurologic examination demonstrated right upper quadrantopia and diminished sensation to light touch and pinprick in the right upper and lower extremities. Otherwise, cranial nerves III–XII were intact. Strength was 5/5 in the bilateral upper and lower extremities, and no diminished sensation was noted on the left side of the patient's body.

## 3 Methods

Given this initial evaluation, his National Institutes of Health Stroke Score (NIHSS) was calculated as 2. A stroke alert was activated, and the neurology team was consulted. The patient underwent computed tomography (CT) angiography of the head and neck, which showed an associated prominent left PCA territory infarct in the distal left P2 segment (see Figures 1A,B and 2A,B). The patient was updated with these findings, and after discussion between the ED and neurology team, 0.25 mg/kg (22 mg) of Tenecteplase was administered. Administration was completed less than 3 h after symptom onset. Within 30 min, the patient's numbness had improved. At the same time, interventional radiology was consulted for potential endovascular intervention; however, the infarct was considered too distal for retrieval. He was subsequently admitted and underwent magnetic resonance imaging, which demonstrated the previously seen acute left PCA distribution infarction (see Figure 1C).

**FIGURE 1:**
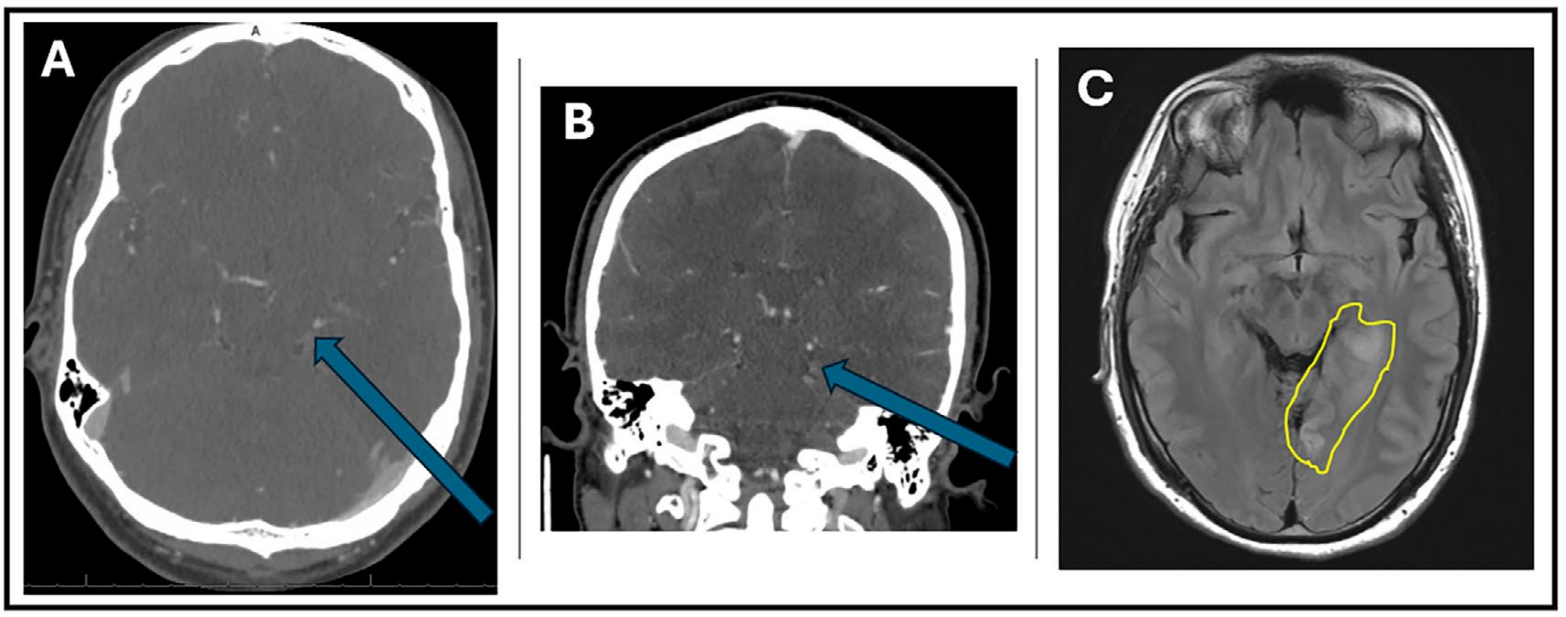
Axial (A) and coronal (B) angiography showing distal left posterior cerebral artery occlusion consistent with infarction, obtained 2.5 h after symptom onset. Blue arrows point to the lesion. Magnetic resonance imaging (T2 Fluid‐Attenuated Inversion Recovery, FLAIR) was obtained following admission (C) demonstrating the same, with the yellow highlighted area demonstrating the area of infarct.

**FIGURE 2:**
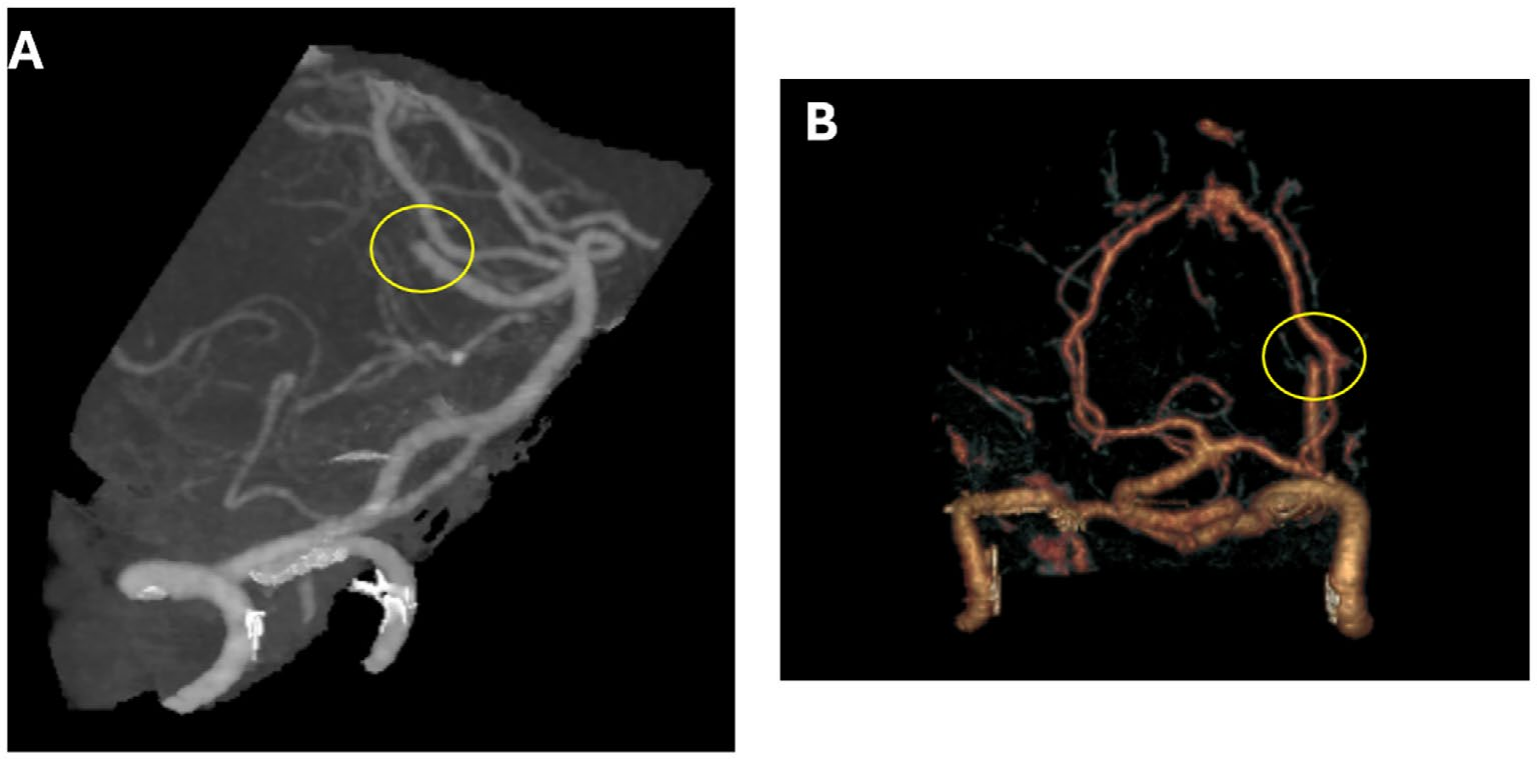
Left‐to‐right (A) and caudal‐to‐cranial (B) views of the three‐dimensional reconstructions obtained during angiography of the patient presented. The circled areas demonstrate the acute left posterior cerebral artery infarction seen prior to reconstructions.

The source of clot formation in this patient included cardiac arrhythmia such as atrial fibrillation, paradoxical embolism from a venous source, coagulopathy, or an infectious or autoimmune process triggering vasculitis, dissection, or direct thrombus formation. There was lower suspicion of arterial atherosclerosis as a thrombotic source given the patient's age and lack of risk factors. A cardioembolic source was considered the most likely. Upon further evaluation, the patient did report he had multiple shoulder dislocations in the past, and the possibility of a dissection related to connective tissue disorder was considered, though none was identifiable on CT angiography of the head and neck. Coagulation studies, including phosphatidyl serine, anticardiolipin, beta‐2 glycoprotein, protein C and S levels, and homocysteine levels, were analyzed and found to be within normal limits. Hemoglobin A1c was 5.1. Bilateral lower extremity ultrasounds were negative for clots. No cardiac arrhythmia was noted on any telemetry or electrocardiogram obtained in the hospital. The patient underwent transthoracic and subsequently transesophageal echocardiography, which demonstrated a large right‐to‐left shunt between the atria, consistent with a patent foramen ovale (PFO).

## 4 Conclusion and Results

Within 24 h of Tenecteplase administration, the patient's symptoms had completely resolved. Apixaban for treatment of presumed DVT and high‐dose rosuvastatin were initiated, and he was discharged. The large PFO was repaired later, and the patient remains in good health as of the last evaluation.

## 5 Discussion

Cerebral vascular accidents (CVAs) are a common cause for acute neurologic change in elderly patients or those who have significant risk factors. They are less common and therefore less readily recognized in young patients who are in good health [5, 6, 7]. By definition, CVAs are sudden focal neurological deficits caused by ischemia (87%) or hemorrhage (13%). Ischemic mechanisms include cardioembolism (e.g., atrial fibrillation), large artery atherosclerosis (e.g., carotid), small vessel occlusion (e.g., lacunar), dissections (e.g., vertebral or cervical arteries), hypoperfusion, and in situ thrombosis. A paradoxical embolism, as suspected in this patient, arises when there is a right‐to‐left shunt in the heart or lungs. This leads to thromboemboli from the venous circulation passing into the arterial circulation and occluding the vascular supply to the brain. PFOs are sometimes found incidentally in patients with significant risk factors and a high likelihood of a more conventional etiology. In these cases, a “Risk of Paradoxical Embolism (RoPE) Score” can be calculated to indicate the likelihood that the stroke was due to the PFO [8]. The patient in this case had a RoPE score of 10 points (patient had a cortical infarct on imaging), which corresponds to an 88% chance the patient's stroke was due to a PFO, with a predicted 2% risk of 2‐year recurrence of stroke or transient ischemic attack. However, while the RoPE score indicates the PFO is the likely conduit for a paradoxical embolism, it does not specify the origin of the thrombus or the trigger for its embolization.

A PFO is a common pathology and is seen in approximately one quarter of the general population [9, 10, 11]. However, about one‐half of patients with cryptogenic strokes have a PFO, which suggests a causal relation. While the patient above had negative scans for deep vein thromboses in his bilateral lower extremities, given his recent car ride, it is theorized that he may have had a subclinical clot that was dislodged during masturbation. The dislodgement may have been by chance or by the physiological changes that may occur during masturbation/sexual activity. A PFO can also cause strokes via thrombosis related to turbulent flow of blood through the shunt itself [1]. Other sites, like the pelvis or upper extremities, may have been the source and were not identified. It should be said that the 2.5‐h car ride is a weak risk factor for clot formation, and there may be other factors at play that were not evaluated. Further compounding the problem is that, in some cases, there can be a significant rate of false negatives among bilateral lower extremity DVT ultrasounds [12].

Autoimmune connective tissue disease can cause thrombotic strokes through vasculitis, dissection, or thromboembolism related to a hypercoagulable state [13, 14, 15, 16]. These were considered for this patient, given that he reported multiple shoulder dislocations, but the hypercoagulability workup was negative.

It is theorized that rapid fluctuations in heart rate and blood pressure during masturbation may have dislodged a subclinical clot in this patient's venous circulation. This passed through his PFO and into the posterior arterial circulation of his brain. Fluctuations in blood pressure and heart rate have been noted during sexual activity, including masturbation [17]. Sexual activity is a risk factor for the spontaneous rupture of cerebral aneurysms due to the associated increase in blood pressure and thus poses a risk for subarachnoid hemorrhage in susceptible patients [18]. While sexual activity itself has not been linked to ischemic stroke, the chronology of the case presented above makes a dislodged thrombus a possible exception, considering that masturbation may reach the level of vigorous activity, which is an accepted trigger [4, 18].

An alternative theory is that a clot had already formed and partially embolized during the patient's previously noted headaches with transient visual disturbances. These possible TIAs may have preceded the full embolization of the clot, resulting in the stroke. This is not the typical pattern, however, as usually only part of the clot embolizes, causing a stroke or pulmonary embolism. As noted above, the act of masturbation may have contributed, but this could have been a progressive condition that would have happened regardless of what the patient was doing. We cannot confirm that masturbation was the cause of this patient's stroke. Still, we present this case as a stroke occurring during masturbation with a theory that it may have contributed to his presentation.

In conclusion, strokes in young, healthy patients are rare events that carry significant morbidity and mortality. Patients presenting with sudden‐onset neurologic deficits should be considered for a possible stroke regardless of age. A patent foramen ovale is implicated in more than half of all cryptogenic strokes among patients ≤ 60 years of age. While other etiologies for potential thrombus creation/dislodgement, including trauma, thrombophilia, and vigorous exercise, have been reported, it is theorized that clot dislodgement during masturbation contributed to this patient's presentation. A detailed history in any patient, regardless of age or identifiable risk factors, may reveal potential sources for thrombus formation and mechanisms for their dislodgement, leading to increased suspicion and recognition of stroke, which is intervenable in the hyperacute setting.

## Author Contributions

**Abdulmuti Saleh:** conceptualization, data curation, project administration, resources, writing – original draft. **Florian Merkle:** conceptualization, data curation, supervision, writing – review and editing. **Ashley Brown:** writing – review and editing. **Michael C. Larkins:** conceptualization, data curation, resources, writing – review and editing. **Michael Tensmeyer:** conceptualization, data curation, writing – review and editing.

## Funding

All authors are required to disclose any and all commercial, financial, and other relationships in any way related to the subject of this article as per ICMJE conflict of interest guidelines (see www.icmje.org). The authors have stated that no such relationships exist.

## Consent

Written informed consent was obtained from the patient for the publication of this case report.

## Conflicts of Interest

M.C.L. reports he is an employee of the United States Air Force, and therefore requires the following disclaimer to be included upon publication of this document:

Disclaimer—The views and opinions expressed in this article/presentation are those of the author(s) and do not necessarily reflect official policy or position of the United States Air Force, Defense Health Agency, Department of Defense, or U.S. Government.

The remaining authors do not report any conflicts of interest.

## Data Availability

The data that support the findings of this study are available on request from the corresponding author. The data are not publicly available due to privacy or ethical restrictions.
